# Drug-induced activation of SREBP-controlled lipogenic gene expression in CNS-related cell lines: Marked differences between various antipsychotic drugs

**DOI:** 10.1186/1471-2202-7-69

**Published:** 2006-10-20

**Authors:** Johan Fernø, Silje Skrede, Audun O Vik-Mo, Bjarte Håvik, Vidar M Steen

**Affiliations:** 1 Dr. Einar Martens' Research Group for Biological Psychiatry and Bergen Mental Health. Research Center, Department of Clinical Medicine, University of Bergen, Norway; 2 Center for Medical Genetics and Molecular Medicine, Haukeland University Hospital, Helse Bergen HF, Norway

## Abstract

**Background:**

The etiology of schizophrenia is unknown, but neurodevelopmental disturbances, myelin- and oligodendrocyte abnormalities and synaptic dysfunction have been suggested as pathophysiological factors in this severe psychiatric disorder. Cholesterol is an essential component of myelin and has proved important for synapse formation. Recently, we demonstrated that the antipsychotic drugs clozapine and haloperidol stimulate lipogenic gene expression in cultured glioma cells through activation of the sterol regulatory element-binding protein (SREBP) transcription factors. We here compare the action of chlorpromazine, haloperidol, clozapine, olanzapine, risperidone and ziprasidone on SREBP activation and SREBP-controlled gene expression (*ACAT2, HMGCR, HMGCS1, FDPS, SC5DL, DHCR7, LDLR, FASN *and *SCD1*) in four CNS-relevant human cell lines.

**Results:**

There were marked differences in the ability of the antipsychotic drugs to activate the expression of SREBP target genes, with clozapine and chlorpromazine as the most potent stimulators in a context of therapeutically relevant concentrations. Glial-like cells (GaMg glioma and CCF-STTG1 astrocytoma cell lines) displayed more pronounced drug-induced SREBP activation compared to the response in HCN2 human cortical neurons and SH-SY5Y neuroblastoma cells, indicating that antipsychotic-induced activation of lipogenesis is most prominent in glial cells.

**Conclusion:**

Our present data show a marked variation in the ability of different antipsychotics to induce SREBP-controlled transcriptional activation of lipogenesis in cultured human CNS-relevant cells. We propose that this effect could be relevant for the therapeutic efficacy of some antipsychotic drugs.

## Background

Schizophrenia is a chronic and serious psychiatric disorder affecting about 1 % of the population worldwide. Several lines of evidence point towards schizophrenia as a neurodevelopmental disorder, and the involvement of myelin- and oligodendrocyte abnormalities in the pathophysiology of the disease has been suggested [1-5]. Myelin is formed by oligodendroglial cells and consists mainly of cholesterol, produced *de novo *in the CNS [6]. Together, cholesterol and apolipoprotein E (ApoE) can serve as a glial growth factor in synaptogenesis [7].

Antipsychotic drugs, such as haloperidol and clozapine, are used to treat and ameliorate the symptoms of schizophrenia and act, at least in part, by blockage of dopamine D2-like receptors in the brain. Antipsychotics are usually classified into two main groups, namely typical (e.g. haloperidol and chlorpromazine) and atypical drugs (e.g. clozapine, olanzapine, risperidone and ziprasidone). The typical antipsychotic drugs are all dopamine D2-receptor antagonists, whereas the atypical drugs generally have a more diverse receptor binding profile, suggesting that other neurotransmitters than dopamine might also be involved in mediating the antipsychotic effect [8]. Compared to typical antipsychotics, atypical drugs usually induce less extrapyramidal side effects and may have improved therapeutic efficacy [9,10]. However, some of the atypical drugs are associated with side effects such as weight gain and other metabolic adverse [11].

In a recent study, we demonstrated that haloperidol and clozapine markedly increase the expression of a cluster of fatty acid- and cholesterol biosynthetic genes in a human glioma cell line (GaMg), mediated via activation of the sterol regulatory element-binding proteins (SREBP) [12]. We proposed that the SREBP-mediated activation of cellular lipogenesis represents a new mechanism of psychotropic drug action, involved in both therapeutic efficacy (through enhanced synthesis of cholesterol in CNS) and the metabolic side-effects (through increased production of fatty acids and triglycerides in peripheral tissues) [12].

SREBPs are produced as two isoforms; SREBP1 (with two splice variants, SREBP1a and -1c) and SREBP2. The SREBP1 and SREBP2 transcription factors are synthesized as inactive 120 kDa precursors in the endoplasmic reticulum (ER). Upon activation, these proteins are translocated to the Golgi apparatus by the sterol sensing SREBP-cleavage-activating-protein (SCAP) where they undergo proteolytical cleavage, releasing a 60–70 kDa fragment that is transcriptionally active and induces the expression of several lipid biosynthetic genes in the nucleus (for review, see [13]). The different SREBP variants overlap in function to some extent [14]. In general, SREBP1c controls the expression of fatty acid biosynthesis genes in various tissues [15], whereas the SREBP1a isoform is predominant in cell cultures, activating both cholesterol and fatty acid biosynthesis genes [16,17]. SREBP2 mainly regulates cholesterol biosynthetic genes both in tissues and in cell culture [18].

In the present study, we have extended our knowledge on lipogenic effects of psychotropic drugs by comparison of six antipsychotics with respect to their dose-related ability to activate SREBP and the expression of SREBP target genes in human glial GaMg cells. We also examined the degree of SREBP activation in three additional CNS-related cell lines, including glial cell lines (astrocytoma CCF-STTG1 cells) and neuronal cell lines (neuroblastoma SH-SY5Y cells and cortical neuronal HCN2 cells). We here report that chlorpromazine, haloperidol, clozapine, olanzapine, risperidone and ziprasidone display marked differences in their ability to stimulate SREBP-controlled cellular lipogenesis, and that this effect is most pronounced in glial cells. We propose that SREBP activation might represent a novel glia-mediated mechanism of action of some antipsychotic drugs.

## Results

### Cell viability tests

The effect on GaMg cell viability was investigated for all six antipsychotic drugs (chlorpromazine, haloperidol, clozapine, olanzapine, risperiodone and ziprasidone), using a range of concentrations from 1 μM to 100 μM (data not shown). Olanzapine had no cell toxic effect at any of the concentrations tested. Up to 25 μM, no statistically significant reduction in cell viability was observed for haloperidol, clozapine and risperidone. Cell viability in response to chlorpromazine exposure (10 μM and 25 μM) was decreased by about 30 % and 50 %, respectively. Ziprasidone reduced cell viability by 30 % and 75 % at 10 μM and 25 μM, respectively, as compared to the control.

### Antipsychotic-induced activation of the SREBP target genes

**Figure 1:**
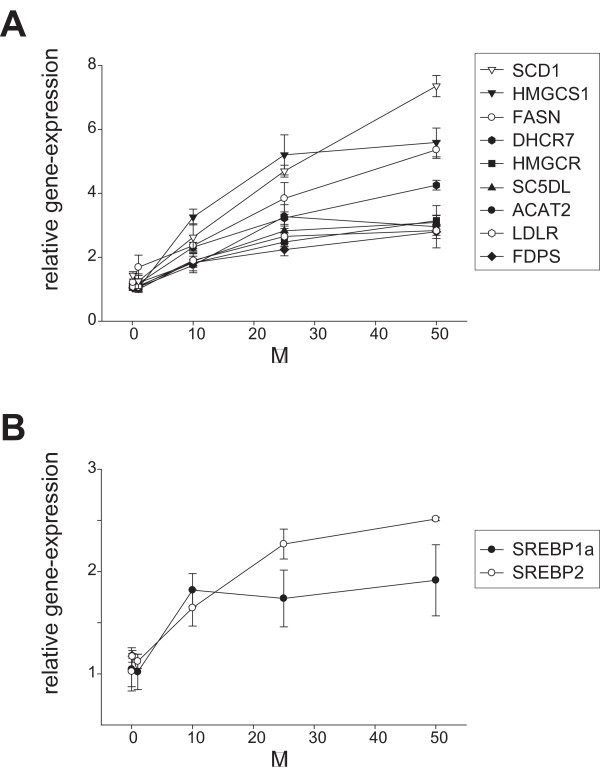
**The effect of clozapine on lipogenic gene expression in GaMg cells**. Gene expression was determined in GaMg cells after exposure of various concentrations of clozapine for 24 hours. The level of expression was measured by quantitative RT-PCR, relative to the expression of the ribosomal gene *P0*. The p-values were obtained by a Dunnett post-hoc test following a one-way ANOVA. Error bars display ± SEM, and the data are representative of three independent parallel experiments. **A**) Relative expression levels of genes involved in cholesterol biosynthesis (filled symbols), cholesterol transport and fatty acid biosynthesis (open symbols). The expression of all genes was significantly increased (fold change at 50 μM clozapine, p-value): stearoyl-CoA desaturase (delta-9-desaturase; *SCD1*) (7.4, p = 0.000008), 3-hydroxy-3-methylglutaryl-CoA synthase 1 (*HMGCS1*) (5.6, p = 0.000009), fatty acid synthase (*FASN*) (5.4, p = 0.000008), 7-dehydrocholesterol reductase (*DHCR7*) (4.3, p = 0.000008), 3-hydroxy-3-methylglutaryl-CoA reductase (*HMGCR*) (3.1, p = 0.00001), sterol-C5-desaturase like (*SC5DL*) (3.1, p = 0.00001), acetyl-CoA acetyltransferase 2 (also termed acetoacetyl-CoA thiolase; *ACAT2*) (3.0, p = 0.00009), low density lipoprotein receptor (*LDLR*) (2.8, p = 0.00001) and farnesyl diphosphate synthase (*FDPS*) (2.8, p = 0.000009). **B**) Relative expression levels of *SREBP1a *(open symbols) and *SREBP2 *(filled symbols). Both *SREBP1a *(2.2-fold, p = 0.02) and *SREBP2 *(2.5-fold, p = 0.0002) were increased by clozapine in a dose-dependent manner.

In an attempt to further understand antipsychotic-induced stimulation of cellular lipogenesis, we examined similarities and differences between several SREBP target genes involved in various aspects of lipid homeostasis. As shown in Fig. 1a, clozapine (0.1 μM – 50 μM) induced a statistically significant dose-dependent transcriptional activation of all nine SREBP target genes examined in cultured GaMg cells. These genes are involved in cholesterol biosynthesis (*ACAT2*, *HMGCS1*, *HMGCR*, *FDPS*, *SC5DL *and *DHCR7*; see legend to Fig. 1 for complete names), cholesterol transport (*LDLR*) and fatty acid biosynthesis (*FASN *and *SCD1*). The degree of maximum activation (obtained by 50 μM clozapine) varied markedly between the different genes, with fold-changes ranging from 2.2 (*FDPS*) to 7.3 (*SCD1*) (Fig. 1a). The expression of *SREBP1*a and *-2 *was readily detected, and the expression levels of both genes increased markedly with increasing clozapine concentrations (Fig 1b). The transcriptional activation was most pronounced for the *SREBP2 *gene. The expression of *SREBP1c *also appeared to be significantly increased by clozapine (results not shown), but due to the low expression levels (Ct-values around 36), the interpretation of the data should be cautious.

### Comparison of different antipsychotic drugs with respect to their SREBP-stimulating effect

In order to compare various drugs with regard to their ability to activate SREBP-mediated gene expression, a selection of SREBP target genes (*HMGCS1*, *HMGCR*, *SC5DL *and *LDLR*) was measured after exposing GaMg cells to various concentrations (0.1, 1, 10 and 25 μM) of chlorpromazine, haloperidol, clozapine, olanzapine, risperidone or ziprasidone (Table 1). Clozapine and haloperidol, and to a lesser extent olanzapine and chlorpromazine, clearly enhanced the transcription of all four SREBP target genes, especially *HMGCS1 *and *HMGCR*. For olanzapine, the stimulating effect was most pronounced at the highest concentration (25 μM), whereas chlorpromazine tended to be less effective at the same concentration, probably due to drug toxicity (see above). Interestingly, risperidone and ziprazidone demonstrated significantly lower ability to activate the expression of the SREBP-controlled genes. For ziprasidone, the limited action could not be overcome by increased concentrations, since the drug then induced a marked reduction in cell viability.

**Figure 2:**
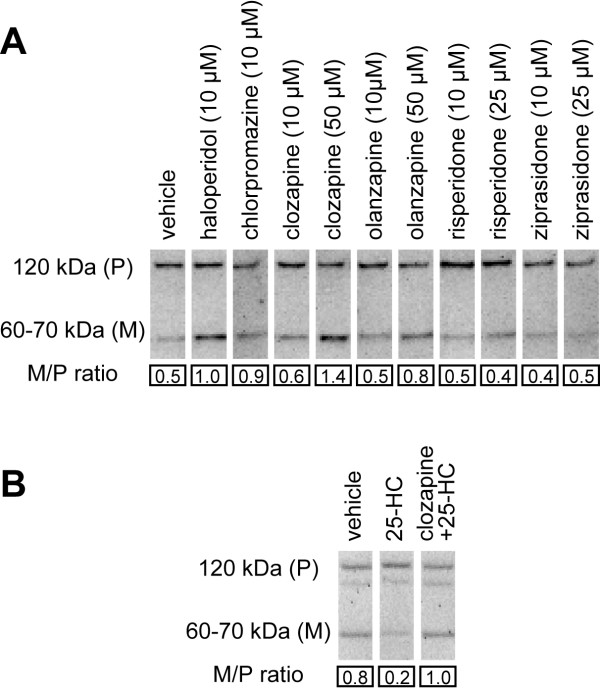
**Proteolytic cleavage of the SREBP2 transcription factor**. A) The semi-quantitative M/P-ratio of the mature transcriptionally active 60–70 kDa fragment (M) and the SREBP2 120 kDa inactive precursor (P) as a measurement of the degree of SREBP2 activation following antipsychotic drug treatment. The results are representative of two independent western blot experiments. In agreement with the gene expression results, haloperidol, chlorpromazine, clozapine and olanzapine all induced marked SREBP2 cleavage. B) The effect of clozapine on 25-hydroxycholesterol (25-HC) -induced repression of endogenous SREBP-2 activity in GaMg cells. Clozapine (30 μM) significantly counteracts the SREBP2 repression induced by 25-HC (1 μg/ml) after 2 hours of incubation. The results are representative of two independent experiments.

The psychotropic-induced transcriptional activation of cellular lipogenesis in GaMg cells is due to an increased proteolytic cleavage of the SREBP transcription factors, especially SREBP2 [12]. In the present study, we therefore examined the effect of the six antipsychotics on proteolytic cleavage and activation of SREBP2 only. In western blot experiments, the semi-quantitative M/P ratio between the mature transcriptionally active 60–70 kDa fragment (M) and 120 kDa precursor protein (P) was used as an indicator of SREBP2 stimulation, as measured after 24 hours of drug exposure in GaMg cells (Fig. 2a). The degree of SREBP2 activation was parallel to the expression level of SREBP2 target genes for all drugs (see Table 1 for comparison). Haloperidol, chlorpromazine, and at higher non-toxic concentrations, clozapine and olanzapine, all induced SREBP2 cleavage markedly above the control level. Risperidone and ziprasidone apparently had less pronounced or no effect on SREBP2 activation.

### Clozapine counteracts the repressive effect of 25-hydroxycholesterol on SREBP activation

To further explore the mechanism behind the drug-induced activation of SREBPs, we investigated whether clozapine could interfere with the effect of 25-hydroxycholesterol (25-HC), a well-known SREBP repressor [19]. The repressive effect of 25-hydroxycholesterol is mediated by its ability to increase cholesterol transport from the cell plasma membrane to the SCAP sterol sensor in the ER [20]. 25-HC (1 μg/ml) markedly reduced basal SREBP2 activity after 2 hours of incubation in cultured GaMg cells (Fig 2b). Co-treatment with clozapine (30 μM) clearly counteracted the repressive effect of 25-HC. This opposing effect was not detectable after longer periods of co-incubation (4–24 hours), probably due to the high potency of 25HC to repress SREBP (results not shown).

### Antipsychotic-induced SREBP activation in the context of therapeutic serum concentrations

**Figure 3:**
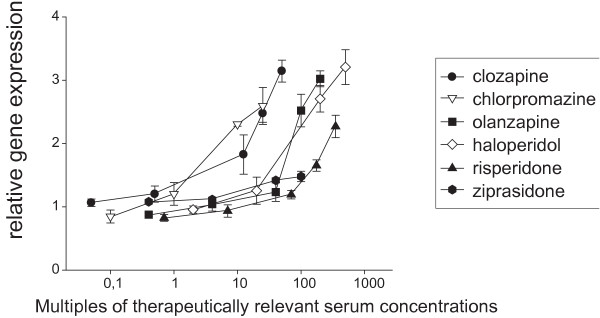
**Dose-response curve in antipsychotic-exposed GaMg cells extrapolated to therapeutically relevant serum levels**. *HMGCR *expression levels changes for each drug, following transformation of the molar concentrations used in the cell culture experiment into multiples of their therapeutically relevant serum levels (see below). Due to effects on cell viability, the highest concentrations of chlorpromazine, haloperidol and ziprasidone in the culturing media was 25 μM. Typical and atypical antipsychotic drugs are denoted with open and filled symbols, respectively. Note the logarithmic scale on the x-axis. The upper therapeutic serum concentration for each drug was set according to the AGNP-TDM expert group consensus guidelines [36]: clozapine 2000 nM, chlorpromazine 1000 nM, olanzapine 250 nM, haloperidol 50 nM, risperidone 140 nM and ziprasidone 250 nM.

In clinical practice, the therapeutically relevant serum levels vary considerably between the different antipsychotic drugs. In order to examine the potential clinical relevance of the drug-induced SREBP activation that was observed in cultured cells, the molar concentrations used in the cell culture experiment were transformed into multiples of the therapeutically relevant serum concentration for each drug (Fig 3; see the legend for further details about the transformation factor). The transformed data illustrate that although all drugs stimulated *HMGCR *expression at some concentrations, only clozapine and chlorpromazine induced significant SREBP activation in the cell cultures at concentrations rather close to their therapeutic serum levels. For the other drugs, the stimulation of gene expression occurred at concentrations far above their therapeutically relevant serum levels. The issue of cell culture concentrations, serum levels and relevant tissue (brain) concentrations of the drugs is further outlined in the Discussion section.

### Comparison of antipsychotic-induced activation of SREBP target genes in different human CNS-relevant cell lines

In order to study whether the antipsychotic-induced SREBP activation is valid in more than one CNS-related human cell line, we examined *HMGCR *expression in astrocytoma CCF-STTG1 cells, neuroblastoma SH-5YSY cells, and cortical neuronal HCN2 cells, all exposed to clozapine or haloperidol for 24 hours. The largest increase in gene expression was observed in the astrocytoma cells, reaching a statistically significant fold change of 5.0 ± 0.2 and 9.4 ± 0.4 with clozapine (25 μM) and haloperidol (25 μM), respectively (Table 2). In contrast, the SH-SY5Y neuroblastoma cells displayed minimal activation of the SREBP transcription system upon drug exposure, with a relative increase in *HMGCR *expression of 1.5 ± 0.1 and 1.4 ± 0.1 by haloperidol (25 μM) and clozapine (25 μM), respectively (Table 2). In the slow growing HCN2 cells, the *HMGCR *gene expression was activated slightly to moderately by the drugs, demonstrating that antipsychotic-induced SREBP activation is not a tumor cell-specific phenomenon. In a pilot experiment (n = 1), we also examined the R-Hi-501 rat hippocampus primary cell culture, which contains both glial and neuronal cells. Incubation with 25 μM of clozapine led to a 2.1-fold increase in HMGCR mRNA. The expression levels of *HMGCS1 *and *FASN *were also tested in these cells and increased 1.6- and 2.1 fold, respectively. The basal expression level of HMGCR was quite similar in all cell types, as measured with real-time PCR, thereby allowing comparison between different cell lines with respect to the effect of drug exposure.

## Discussion

In this study, we have extended our initial findings with haloperidol and clozapine by demonstrating that antipsychotic drugs in general enhance the expression of SREBP-controlled lipid biosynthetic genes in a concentration-dependent manner in cultured glial cells, with less pronounced stimulation in neuroblastoma- and cortical neuronal cells. The degree of SREBP activation, however, varies considerably between the different antipsychotics.

The mechanisms by which the antipsychotic drugs activate the SREBP system could, in principle, involve receptor-dependent and -independent processes. All antipsychotic drugs block dopamine D2-like receptors (DRD2, DRD3 and DRD4), but with marked differences in their affinities [21,22]. In addition, they also vary widely in their antagonistic binding to other neurotransmitter receptors, e.g., 5-HT and histamine H1 receptors [22]. In the present dose-response comparison, haloperidol and clozapine were the most potent activators of SREBP-controlled gene expression on the basis of their molar concentrations in the cell cultures, whereas risperidone and ziprasidone were minimally effective. Chlorpromazine and olanzapine had intermediate effects. These data imply that the SREBP activation is neither linked to the receptor profiles nor the therapeutic classification (typical vs. atypical) of the drugs, since there is no apparent relationship between these properties and their SREBP-stimulating effect.

This assumption is supported by a recent study [23], in which we demonstrated that several antidepressant drugs (especially tricyclic antidepressants), but not mood stabilizers (carbamazepine and valproate), activate the SREBP system in cultured glial cells in a similar manner to the antipsychotics. This fact makes is likely that the drug-induced SREBP activation is related to some shared chemical property of these psychotropic compounds. Due to the different receptor-binding properties of these drugs it is likely that their shared SREBP-activating effect is mediated via a receptor-independent mechanism of action. All of the antipsychotics and antidepressants that we have investigated are cationic amphiphiles. Such substances have previously been shown to increase the synthesis and accumulation of total cellular cholesterol levels via a mechanism involving a reduction of cholesterol levels in the endoplasmic reticulum (ER), which is the sterol-sensing compartment in the cell [20,24]. SREBP activation is controlled by the sterol-sensitive SREBP-cleavage-activating-protein (SCAP) that is located in the ER [25]. When ER-cholesterol is depleted, SCAP undergoes a conformational change that promotes translocation of the SREBP/SCAP complex to the Golgi, representing the inital step in SREBP activation. Both cholesterol and a hydroxylated derivative of cholesterol, 25-OH-cholesterol (25-HC), inhibit the SREBP system by affecting the function of the SCAP protein [26]. It is therefore interesting to note that clozapine was able to counteract the SREBP-repressing effect of 25-HC, which indicates that the effect of antipsychotic drugs on SCAP/SREBP activation is the opposite of cholesterol and 25-HC. This contrasts *in vitro *data from a recent study demonstrating that some cationic amphiphiles (including clozapine, haloperidol and chlorpromazine) mimic the effect of cholesterol on SCAP conformation [27]. However, in line with our results, this study could not reproduce the cholesterol-mimicking effect of cationic amphiphiles when tested in cultured cells [27].

We propose that drug-induced transcriptional activation of cholesterol biosynthesis in the brain could represent a new mechanism of action for some psychotropic drugs. Interestingly, the SREBP-stimulating effect is shared by several different antipsychotics and antidepressants, demonstrating a common molecular mode of action. This effect cannot be related to the group-specific antipsychotic- or antidepressant properties of the drugs. Instead, it might be linked to some common symptoms or deficits that are present in both schizophrenia and major affective disorders (e.g., cognitive dysfunctions). This possibility is further underscored by the therapeutic breadth of these classes of drugs, with antipsychotic drugs frequently used in treatment of depressed patients and vice versa.

Involvement of myelin- and oligodendrocyte (glial) abnormalities has been indicated in the etiology of both schizophrenia and bipolar disorder [1-5]. The myelination process requires intact cholesterol biosynthesis [6,28], and during CNS development, the expression of cholesterol biosynthesis genes is enhanced [29]. Furthermore, malfunction of synaptic processes, including reduced dendritic spine density, has been proposed in the pathophysiology of schizophrenia [30-33]. Cholesterol, together with ApoE, can act as a glia-derived growth factor supporting the formation of synapses in culture [7]. In line with this, cholesterol has recently been demonstrated as essential for dendrite maturation, the rate-limiting step in glia-induced synaptogenesis, and is required for continuous synapse development in cultures [34]. Interestingly, drug-induced SREBP activation was most evident in the glial-like GaMg glioma- and CCF-STTG1 astrocytoma cell lines, and less pronounced in the HCN2 cortical neuron cells and in a primary cell culture from rat hippocampus. Lipogenic activation was almost absent in SH-SY5Y neuroblastoma cells. These data are in agreement with the fact that in the CNS, the majority of cholesterol is produced *de novo *by glial cells [6]. Cultured neurons have a poorly developed machinery for cholesterol biosynthesis and rely on glia-derived cholesterol for synaptogenesis [35]. It is thus conceivable that drug-induced SREBP-controlled activation of glial cholesterol biosynthesis in the brain represents a receptor-independent therapeutic mechanism of action and provides essential building blocks to the myelination process or synaptogenesis.

A critical issue is whether the SREBP-stimulating effect observed in cultured cells also occurs during drug treatment in the clinical setting. Although any interpretation of the clinical relevance of cell culture data is difficult, the relationship between *in vitro *and *in vivo *(therapeutically relevant) concentrations of the drugs requires special attention. The investigated drugs are clinically efficacious at highly different ranges of serum concentrations [36]. In an attempt to highlight the possible clinical impact of their SREBP-activation, we transformed the molar concentrations of each drug (as used in the GaMg cell culture experiments) into multiples of the therapeutically relevant upper serum concentrations of the corresponding drug (as determined from the AGNP-TDM expert group consensus guidelines) [36]. By this approach, clozapine and chlorpromazine appeared to significantly activate the SREBP system in the cultured cells at concentrations that were 5–10-fold above their therapeutic serum levels. In contrast, the concentration of haloperidol necessary to induce this lipogenic effect in the cultured cells was about 200 times higher than the clinically relevant serum level, and ziprasidone hardly activated the SREBP pathway at all. It is important to bear in mind that many psychotropic drugs are highly lipophilic, leading to enrichment in lipid-rich tissues. Levels of haloperidol and clozapine have been demonstrated to be 10–30 times higher in the CNS compared to the corresponding serum concentration [37,38]. Taken together, these data indicate that SREBP-activation by clozapine and chlorpromazine might occur in the brain when serum levels are within their therapeutically relevant range, whereas the SREBP activation observed for haloperidol would be expected to be of minor clinical relevance. Interestingly, clozapine has been described as a drug with superior therapeutic efficacy [39,40], with positive effects in otherwise treatment-refractory patients. However, a similar supremacy has not been shown for chlorpromazine, the other apparent potent SREBP-activating drug. Finally, it is uncertain how large an *in vivo *response would need to be in order to be of any clinical relevance, since even minor changes in cellular lipid biosynthesis *in vivo *could have marked clinical effects. Caution should always be taken when making inferences from cell cultures to the clinical setting, and further studies are indeed warranted to draw more reliable conclusions about the clinical impact of our results.

## Conclusion

We here extend our initial studies with haloperidol and clozapine by demonstrating that antipsychotic drugs in general stimulate SREBP-controlled gene expression in several CNS-relevant cell lines, but with marked differences between the various drugs and types of cells. We have previously suggested that the psychotropic-induced SREBP activation is a glial-mediated mechanism of drug action. This proposal is supported by our present results, demonstrating that the lipogenic effect is apparently more pronounced in glial than in neuronal cell lines. We hypothesize that drug-induced transcriptional activation of cholesterol biosynthesis in the brain represents a receptor-independent mechanism of therapeutic action of some antipsychotic drugs, possibly by providing essential building blocks for the myelination process and synaptogenesis. Further studies on gene expression changes and lipid parameters in blood samples from drug-treated patients and brain tissue from animal models should be undertaken.

## Methods

### Cell cultures

The human glioma GaMg cell line was kindly provided by an in-house source [41]. The R-Hi-501 rat hippocampal primary cell culture was purchased from Cambrex (Cambrex Biosciences, USA). All other cell lines were purchased from ATCC (LGC Promochem, UK). GaMg human glioblastoma cells were cultured in Dulbecco's Modified Eagle Medium (DMEM) containing 2 mM L-glutamine, 4.5 g/L glucose and 10 % fetal bovine serum (FBS). CCF-STTG1 human astrocytoma cells were grown in RPMI 1640 medium containing 2 mM L-glutamine 1.5 g/L sodium bicarbonate, 4.5 g/L glucose, 10 mM HEPES, 1.0 mM sodium pyruvate and 10 % FBS. SH-SY5Y human neuroblastoma cells were cultured in Eagle's Minimum Essential Medium (EMEM) containing 1.5 g/L sodium bicarbonate, 1.0 mM sodium pyruvate, 0.1 mM non-essential amino acids (NEAA), 2 mM L-glutamine and 10 % FBS. HCN2 human cortical neuron cells cells (derived from cortical tissue removed from a patient undergoing hemispherectomy for intractable seizures associated with Rasmussen's encephalitis) were grown in DMEM medium containing 1.5 g/L sodium-bicarbonate, 4.5 g/L glucose and 4 mM L-glutamine and 10 % FBS. R-Hi-501 rat hippocampal cells were grown in Neurobasal Medium containing 2 mM L-Glutamine and 2 % B27 supplement. GaMg, CCF-STTG1 and SH-SY5Y cells were cultured on 6-well plates (TPP, Switzerland) in monolayer to high confluence. HCN2 were cultured on laminin/poly-D-lysine coated 6-well plates (Becton Dickinson, USA) at high confluence. R-Hi-501 cells were grown in poly-D-lysine and laminin coated plates, prepared according to the manufacturers recommendations. All cells were incubated in the presence of 100 U/ml Penicillin-Streptomycin Mixture (Cambrex Biosciences) in 5 % CO_2 _at 37°C. The cell lines were cultured for 1–2 days before drug exposure and experiments were performed in triplicate, with the exception of the R-Hi-501 cells (n = 1).

### Drug exposure and cell viability testing

For examination of the effects on cell viability for haloperidol (Janssen-Cilag, Belgium), chlorpromazine (Sigma-Aldrich, USA), clozapine (Sigma-Aldrich), olanzapine (Toronto Research Chemicals, Canada), risperidone (Sigma-Aldrich) and ziprasidone (Toronto Research Chemicals), we used the Cell Proliferation Reagent WST-1 (Roche Diagnostics, Germany). GaMg cells were spread out with a density of 6000 cells/well (96 multi-well plates) and exposed to 1, 5, 10, 25, 50 and 100 μM of the respective drug for 24 hours. Cell viability in response to drug exposure was compared to the growth of vehicle-exposed (lactic acid, 6 μg/ml) cells as well as to a cell density gradient created by plating out 3000, 4000, 6000, 8000 or 10000 GaMg cells per well. The WST-1 analysis was performed in accordance with the supplier's recommendations. All experiments were initiated by replacement of the pre-exposure media with fresh media containing vehicle control (lactate acid) or the respective drug. All experiments were performed in quadruplicate.

### RNA extraction, cDNA synthesis and real-time PCR analysis

After drug exposure, cells were trypsinated and total RNA was extracted using the ABI PRISM™ 6100 Nucleic Acid PrepStation (Applied Biosystems, USA). RNA quantity and quality was measured on the NanoDrop^® ^ND-1000 spectrophotometer (NanoDrop Technologies, USA). cDNA was synthesized using the TaqMan Reverse Transcription (RT) reagents (Applied Biosystems) and used as template for real-time PCR analysis on an ABI Prism 7900^HT ^sequence detector system (Applied Biosystems) with SYBR-green (Eurogentec, Belgium) as detector, as previously described [12]. Each sample was run in triplicate. The relative gene expression levels were determined with the comparative ΔCt-method [42] and normalized relative to the ribosomal protein *P0 *gene. Similar results were obtained in a pilot study when normalizing relative to *GAPDH *and *B-actin*.

### Immunoblot analysis

GaMg cells were seeded into 6-well plates and left to adhere over night. To reduce the endogenous SREBP activity, the medium was supplemented to contain 20 % FBS three hours prior to addition of the drugs or vehicle. The cells were grown in the same medium for 24 hours during drug exposure. In order to terminate the experiments, cell cultures were washed twice with cold PBS before adding RIPA lysis buffer (15 mM NaCl, 50 mM TRIS, 0.5 % sodium deoxycholate, 1 % Np-40 and 0.1 % SDS) containing *Complete *protease inhibitor (one tablet per 10 ml RIPA; Roche Diagnostics, USA). To achieve complete lysis, the cell suspensions were kept in the RIPA-containing buffer for 30 min at 4°C, before storage at -20°C.

For Western blotting, the samples were thawed and centrifuged in an Eppendorf 5415D microcentrifuge (Eppendorf, USA) at 16000 × *g *for 10 minutes. Protein content was determined from the supernatant using the Bio-Rad *DC *(detergent compatible) Protein Assay (Bio-Rad Laboratories, USA). Immunoblotting was performed by the Western Breeze system (Invitrogen, USA): 10 μg of total protein from each sample was separated on NuPAGE 4–12 % Bis-Tris Gels and blotted onto Invitrolon™ PVDF membranes. The membranes were incubated overnight at 4°C with 1.5 μg/ml purified monoclonal anti-human SREBP2 mouse antibody (IgG-1C6, BD Biosciences Pharmingen, USA), and probed with alkaline phosphatase-conjugated goat anti-mouse secondary antibody solution (Invitrogen) for 1 hour. Chemiluminescence detection was performed according to manufacturer's instructions (Invitrogen). The SREBP2 activation was semi-quantified as the signal intensity of the mature (M) active 60–70 kDa SREBP fragment divided by the signal intensity of the inactive 120 kDa precursor (P) protein, annotated as the "M/P-ratio". Signal intensity measurements were performed with computer-assisted densitometry and image scanning using the Fuji Las-1000 luminescent image analyzer (Fuji, Japan) and the Image Gauge v4.0 software (Fuji).

### Statistical analysis

Relative gene expression values were analyzed by one-way analysis of variance (ANOVA), when not stated otherwise. When the ANOVA test indicated statistical significance, a Dunnett post-hoc test was applied to determine the concentrations where the change in gene expression was statistically different from the vehicle-treated control. In experimental setups with only few comparisons, the student's t-test was applied. All statistical tests were conducted with Statistica^® ^software version 6.0 (StatSoft, USA).

## Authors' contributions

JF was responsible for the design of the study, participated in the real-time PCR and immunoblot analyses, carried out statistical analysis and drafted the manuscript. SS was involved in the design of the study, participated in the cell culture work and real-time PCR, and contributed to the writing process. BH was involved in the design of the study, participated in cell culture work, and contributed in drafting the manuscript. AOVM participated in design of the study, developed a real-time PCR analysis protocol, and contributed to the writing process. VMS conceived of the study, participated in its design, coordination and data analysis, and helped to draft the manuscript. All authors have read and approved the final manuscript.

## Figures and Tables

**Table 1:** Dose response relationships for antipsychotic drug-induced changes in the expression of SREBP target genes

	***HMGCR***	***HMGCS1***	***SC5DL***	***LDLR***
**chlorpromazine**				
0.1 μM	0.8 ± 0.1	1.0 ± 0.1	1.0 ± 0.09	1.0 ± 0.05
1.0 μM	1.2 ± 0.1	1.8 ± 0.2*	1.2 ± 0.1	1.7 ± 0.3*
10 μM	2.3 ± 0.03***	3.5 ± 0.3***	1.4 ± 0.1***	1.3 ± 0.05*
25 μM	2.6 ± 0.2***	2.3 ± 0.1**	1.3 ± 0.07**	1.0 ± 0.3
**haloperidol**				
0.1 μM	1.0 ± 0.06	1.1 ± 0.1	1.2 ± 0.02	1.1 ± 0.03
1.0 μM	1.3 ± 0.2	1.7 ± 0.3	1.5 ± 0.1**	1.5 ± 0.1
10 μM	2.7 ± 0.2***	4.1 ± 0.5**	3.3 ± 0.07***	2.3 ± 0.2***
25 μM	3.2 ± 0.3***	3.8 ± 0.8*	3.4 ± 0.2***	2.8 ± 0.3***
**clozapine**				
0.1 μM	1.1 ± 0.06	1.1 ± 0.03	1.0 ± 0.01	1.1 ± 0.02
1.0 μM	1.2 ± 0.1	1.0 ± 0.01	1.0 ± 0.02	1.2 ± 0.08
10 μM	1.8 ± 0.3*	3.3 ± 0.2***	1.9 ± 0.1*	1.7 ± 0.2**
25 μM	2.5 ± 0.1***	5.2 ± 0.6***	2.8 ± 0.4***	2.3 ± 0.3***
**olanzapine**				
0.1 μM	0.9 ± 0.03	0.9 ± 0.09	0.8 ± 0.03	1.0 ± 0.1
1.0 μM	1.0 ± 0.1	1.6 ± 0.3	0.9 ± 0.06	1.0 ± 0.05
10 μM	1.2 ± 0.1	1.7 ± 0.2	1.2 ± 0.09	1.3 ± 0.1
25 μM	2.5 ± 0.3*	3.6 ± 0.04***	1.8 ± 0.3**	2.2 ± 0.3**
**risperidone**				
0.1 μM	0.8 ± 0.05	0.9 ± 0.1	0.8 ± 0.01	0.9 ± 0.05
1.0 μM	0.9 ± 0.1	1.0 ± 0.1	0.9 ± 0.01	1.0 ± 0.08
10 μM	1.2 ± 0.07	1.2 ± 0.05	1.1 ± 0.09	1.2 ± 0.1
25 μM	1.7 ± 0.09**	1.8 ± 0.09	1.6 ± 0.2	1.5 ± 0.07
**ziprasidone**				
0.1 μM	1.1 ± 0.02	1.1 ± 0.05	1.2 ± 0.04	1.1 ± 0.03
1.0 μM	1.1 ± 0.02	1.1 ± 0.1	1.2 ± 0.1	1.1 ± 0.03
10 μM	1.4 ± 0.04*	1.5 ± 0.05*	1.5 ± 0.2	1.3 ± 0.04
25 μM	1.5 ± 0.08*	1.3 ± 0.08*	1.7 ± 0.03**	1.5 ± 0.04

The expression levels of *HMGCR*, *HMGCS1*, *SC5DL *and *LDLR *were measured by quantitative RT-PCR in cultured GaMg glioma cells, determined relative to the expression of the ribosomal gene *P0*. The fold change is given relative to the vehicle-treated control. The statistical significance was denoted as follows: *p < 0.05, **p < 0.01, ***p < 0.001. The fold changes (mean ± SEM) are representative of three independent parallel experiments and measured relative to vehicle treated control

**Table 2:** *HMGCR *gene expression changes in human astrocytoma and neuroblastoma cell lines

	**Haloperidol**		**Clozapine**	
**Cell type**	10 μM	25 μM	10 μM	25 μM

SH-SY5Y	1.3 ± 0.06*	1.5 ± 0.1*	0.9 ± 0.02	1.4 ± 0.1*
CCF-STTG1	5.3 ± 0.6**	9.3 ± 0.4***	1.5 ± 0.2*	5.0 ± 0.2***
HCN2	1.2 ± 0.03*	ND	ND	2.4 ± 0.2 **

Human CCF-STTG1 astrocytoma and SH-SY5Y neuroblastoma cells were exposed to haloperidol and clozapine (10 μM and 25 μM of each drug), and human cortical neuron HCN2 cells were exposed to haloperidol (10 μM) and clozapine (25 μM). The *HMGCR*-expression was determined relative to the expression of *P0 *in all cell lines. For CFF-STTG1 and SH-SY5Y, statistical significance of drug-induced *HMGCR *expression was determined by the Dunnetts post-hoc test that correct for multiple testing, as described in Materials and methods. For the HCN2-cells that had only two comparisons, the student's t-test was applied. Each statistical comparison was performed between drug treated cells and vehicle treated control. Significance was denoted as follows: *p < 0.05, **p < 0.01, ***p < 0.001. The fold changes (mean ± SEM) are representative of three independent parallel experiments and measured relative to vehicle treated control. ND: Not determined.
